# Effect of Ultraviolet Irradiation on Polystyrene Containing Cephalexin Schiff Bases

**DOI:** 10.3390/polym13172982

**Published:** 2021-09-02

**Authors:** Anaheed A. Yaseen, Emaad T. B. Al-Tikrity, Emad Yousif, Dina S. Ahmed, Benson M. Kariuki, Gamal A. El-Hiti

**Affiliations:** 1 Department of Chemistry, College of Science, Tikrit University, Tikrit 34001, Iraq; ch@sc.nahrainuniv.edu.iq (A.A.Y.); emaad1954@tu.edu.iq (E.T.B.A.-T.); 2 Department of Chemistry, College of Science, Al-Nahrain University, Baghdad 64021, Iraq; emad.yousif@nahrainuniv.edu.iq; 3 Department of Medical Instrumentation Engineering, Al-Mansour University College, Baghdad 64021, Iraq; dina.saadi@muc.edu.iq; 4 School of Chemistry, Cardiff University, Main Building, Park Place, Cardiff CF10 3AT, UK; kariukib@cardiff.ac.uk; 5 Department of Optometry, College of Applied Medical Sciences, King Saud University, P.O. Box 10219, Riyadh 11433, Saudi Arabia

**Keywords:** cephalexin, Schiff bases, photostability, photodegradation, ultraviolet irradiation absorbers, weight loss

## Abstract

The scale of production of polystyrene has escalated in the recent past in order to meet growing demand. As a result, a large quantity of polystyrene waste continues to be generated along with associated health and environmental problems. One way to tackle such problems is to lengthen the lifetime of polystyrene, especially for outdoor applications. Our approach is the synthesis and application of new ultraviolet photostabilizers for polystyrene and this research is focused on four cephalexin Schiff bases. The reaction of cephalexin and 3-hydroxybenzaldehyde, 4-dimethylaminobenzaldehyde, 4-methoxybenzaldehyde, and 4-bromobanzaldehyde under acidic condition afforded the corresponding Schiff bases in high yields. The Schiff bases were characterized and their surfaces were examined. The Schiff bases were mixed with polystyrene to form homogenous blends and their effectiveness as photostabilizers was explored using different methods. The methods included monitoring the changes in the infrared spectra, weight loss, depression in molecular weight, and surface morphology on irradiation. In the presence of the Schiff bases, the formation of carbonyl group fragments, weight loss, and decrease in molecular weight of polystyrene were lower when compared with pure polystyrene. In addition, undesirable changes in the surface such as the appearance of dark spots, cracks, and roughness were minimal for irradiated polystyrene containing cephalexin Schiff bases. Mechanisms by which cephalexin Schiff bases stabilize polystyrene against photodegradation have also been suggested.

## 1. Introduction

Plastics have many valuable applications including in the production of medical devices, electronics, construction materials, automobiles, packaging, bottles, and toys [1]. In 2018, around 360 million tons of plastics were manufactured to meet global demand [2]. Polystyrene (PS) with high molecular weight is the plastic most in demand. PS is cheap to produce, hard, light, colorless—but can be colored—durable, and resists heat, acid, and alkali [3]. It has numerous applications that range from large equipment to food packaging [4,5]. Solid PS is recyclable but foams used in insulation are not [6]. The crystallinity of PS is controlled by the arrangement of phenyl groups [7]. A random arrangement of phenyl groups leads to atactic PS which is amorphous and of commercial importance. An alternating arrangement of phenyl groups across the polymeric chains leads to the formation of syndiotactic PS which is crystalline [4]. PS has some significant disadvantages such as high solubility in chlorinated solvents, flammability, and resistance to biodegradation. In addition, PS suffers from photodecomposition when exposed to ultraviolet (UV) radiation in the presence of an oxygen source [8,9]. Photodecomposition affects the physical and chemical properties of PS leading to brittleness and stiffness, formation of cracks, and coloration mainly due to cross-linking, resulting in a material with inferior properties [10,11]. It is therefore clear that the photostability needs to be enhanced in order to increase the lifetime of PS in outdoor applications. 

Various additives such as stabilizers, plasticizers, and colorants have been added to the PS matrix to enhance its photostability against photooxidation and photodegradation [12,13,14]. Ideally, additives should be harmless to the environment, not cause a change in color, are not volatile and are compatible with the PS. Additives may be in the form of powders, flakes, beads, or spheres and may act as antioxidants, UV absorbers, energy quenchers, radical decomposers, and flame retardants [15]. The most common PS additives include heterocycles [16,17,18,19], aromatics [20,21], Schiff bases [22], polyphosphates [23], and metal complexes [24]. Aromatic residues in the skeleton of additives play an important role in the improvement of resistance to harsh conditions and oxidants.

Recently, we have reported the use of various Schiff bases as additives for poly(vinyl chloride) (PVC) [25,26,27,28,29,30]. In addition, Schiff bases have the potential to be used as photo-initiators for photopolymerization [31,32]. In continuation of our research, the current study reports the synthesis and use of several cephalexin Schiff bases as additives to enhance the photostability of PS when exposed to UV radiation. Cephalexin is a β-lactamase antibiotic that is mainly used to treat Gram-positive bacteria [33]. It is a solid with a high melting point, is very stable, and contains an aromatic moiety as well as heteroatoms (sulfur, oxygen, and nitrogen). The synthesized cephalexin Schiff bases contain an additional aromatic unit (aryl ring) and heteroatoms (nitrogen, oxygen, and bromine) from the aryl aldehydes. The cephalexin Schiff bases, therefore, meet the structural requirements needed to be PS photostabilizers. We report here the successful synthesis of several cephalexin Schiff bases and their effective use as PS photostabilizers.

## 2. Materials and Methods

### 2.1. General

Polystyrene (Mw = 250,000), cephalexin, aryl aldehydes, chloroform (CHCl_3_), and glacial acetic acid (AcOH) were purchased from Merck (Schnelldorf, Germany). The carbon, hydrogen, nitrogen, and sulfur content were determined on a Vario EL III elemental analyzer (Elementar Americas, Ronkonkoma, NY, USA). Fourier transform infrared (FTIR; 400–4000 cm^−1^) spectra were recorded on a Jasco FTIR-8400 spectrometer (Jasco, Tokyo, Japan). ^1^H (500 MHz) and ^13^C NMR (125 MHz) spectra were recorded on a Bruker DRX-500 NMR spectrometer (Bruker, Zürich, Switzerland) in deuterated dimethyl sulfoxide (DMSO-*d*_6_). UV irradiation (light intensity of 6.43 × 10^−9^ ein dm^−3^ s^−1^ and λ_max_ of 365 nm) of the PS films was performed at 25 °C using an accelerated weather-meter QUV tester (Q-Panel Company, Homestead, FL, USA). The surface of cephalexin Schiff bases was examined by scanning electron microscopy (SEM) using an Inspect S50 microscope (FEI Company, Czechia, Czech Republic). Optical inspection of the PS surface was carried out using a Meiji Techno microscope (Tokyo, Japan). A SIGMA 500 VP microscope (ZEISS Microscopy, Jena, Germany) was used for field emission Scanning electron microscopy (FESEM), and a Veeco 70 instrument (Veeco Instruments Inc., Plainview, NY, USA) for the atomic force microscopy (AFM). The samples were dried using a SQ-15-VAC-16 vacuum oven (MRC Laboratory-Instruments, Essex, UK).

### 2.2. Synthesis of Cephalexin Schiff Bases ***1**–**4***

**Scheme 1:**
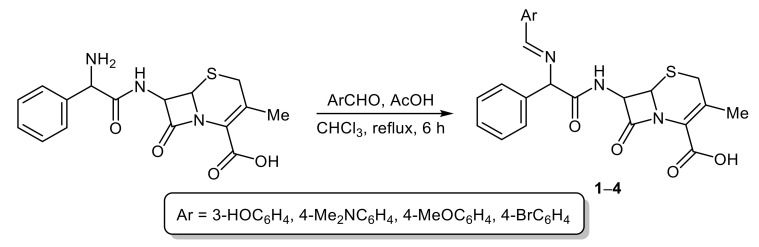
Synthesis of cephalexin Schiff bases **1**–**4**.

A stirred mixture of cephalexin (1.83 g, 5.0 mmol) and an aryl aldehyde (5.0 mmol) in boiling CHCl_3_ (25 mL) containing AcOH (0.5 mL) was refluxed for 6 h (Scheme 1). The mixture was cooled down to room temperature and the solid formed was collected through filtration, washed with CHCl_3_ (10 mL), and dried to give the corresponding Schiff bases **1**–**4** in high yields (Table 1).

### 2.3. Preparation of PS Films

To a stirred solution of PS (5.0 g) in CHCl_3_ (100 mL) at 25 °C, a cephalexin Schiff base (25 mg) was added. The homogenous mixture was stirred at 25 °C for 2 h then poured onto glass slides containing holes that have a thickness of ~40 μm. The PS films produced were left to dry in a vacuum oven for 24 h. The PS films were then irradiated with UV light (λ_max_ = 365 nm) at 25 °C.

## 3. Results and Discussion

### 3.1. Synthesis of Cephalexin Schiff Bases ***1**–**4***

Condensation of an equimolar mixture of cephalexin and 3-hydroxybenzaldehyde, 4-dimethylaminobenzaldehyde, 4-methoxybenzaldehyde, and 4-bromobanzaldehyde in boiling CHCl_3_ under acidic condition gave the corresponding Schiff bases **1**–**4** (Scheme 1) in 70%–82% yields (Table 1). The elemental composition of **1**–**4** was confirmed by the elemental analyses (Table 1). The FTIR spectra of **1**–**4** showed the appearance of absorption bands corresponding to the OH (3320–3489 cm^−1^), NH (3207–3288 cm^−1^), and CH=N (1568-1643 cm^−1^) groups and strong absorption bands due to the carbonyl groups (amide and acid) (Table 2). Some other selected FTIR absorption bands for cephalexin Schiff bases **1**–**4** are also shown in Table 2.

The ^1^H NMR spectra of **1**–**4** showed the appearance of the azomethine proton (CH=N) as a singlet signal in the 8.64–9.31 ppm region (Table 3) which confirms the formation of cephalexin Schiff bases. In addition, they showed the presence of two exchangeable singlets that appeared in the 12.60–13.19 ppm and 7.92–9.63 ppm regions that correspond to the carboxylic and NH protons, respectively. Moreover, the ^1^H NMR spectra of **1**–**4** showed the characteristic two doublets for the CH_2_ protons (which appear separately) and for the CH–CH protons within the lactam ring along with protons from the aromatic moieties. The ^13^C NMR spectra of **1**–**4** (Table 4) showed the presence of all carbons. Four peaks appeared highly downfield corresponding to the carbonyl and the CH=N carbons.

### 3.2. SEM of Cephalexin Schiff Bases ***1**–**4***

**Figure 1:**
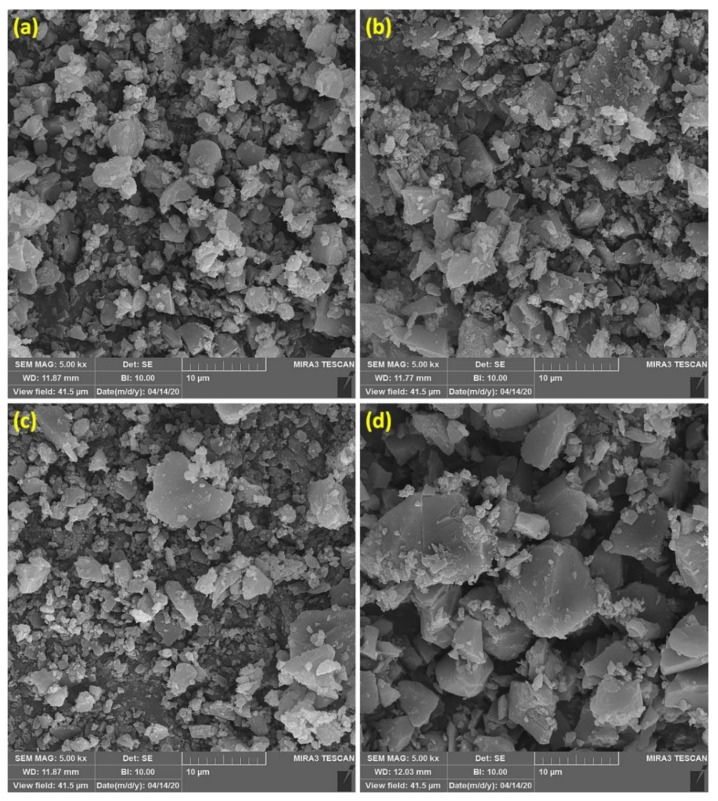
SEM images of cephalexin Schiff bases (**a**) **1**, (**b**) **2**, (**c**) **3**, and (**d**) **4**.

Cephalexin Schiff bases **1**–**4** were characterized further by examining their surfaces using SEM in which an electron beam is used to produce an image [34]. Figure 1 shows that the Schiff bases have smooth, homogeneous, and regular surfaces. The particles size was 40.6–56.7 nm for Schiff base **1**, 40.0–49.8 nm for **2**, 29.5–39.0 nm for **3**, and 42.3–47.2 nm for **4**.

### 3.3. Effects of UV Irradiation on FTIR Spectra

**Figure 2:**
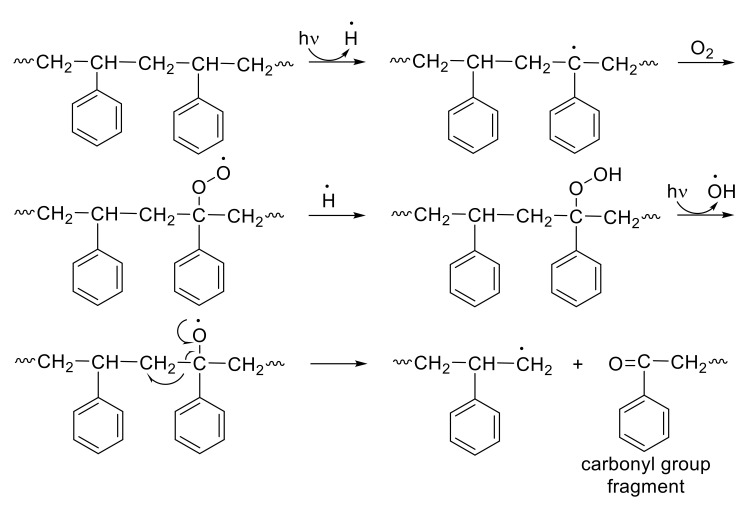
Formation of carbonyl group fragments due to PS photooxidation.

Photooxidation due to UV irradiation in the presence of oxygen has harmful effects (e.g., discoloration and loss of mechanical properties) on the properties of PS [35,36,37]. Such a process leads to the formation of PS free radicals and in the presence of oxygen, oxygenated free radicals are produced (Figure 2). The result is the formation of small fragments containing different functional groups with the most common residues containing the carbonyl group. Therefore, the effect of irradiation on PS can be assessed using IR spectroscopy by monitoring the intensity of the absorption peak corresponding to the carbonyl group [38,39]. 

**Figure 3:**
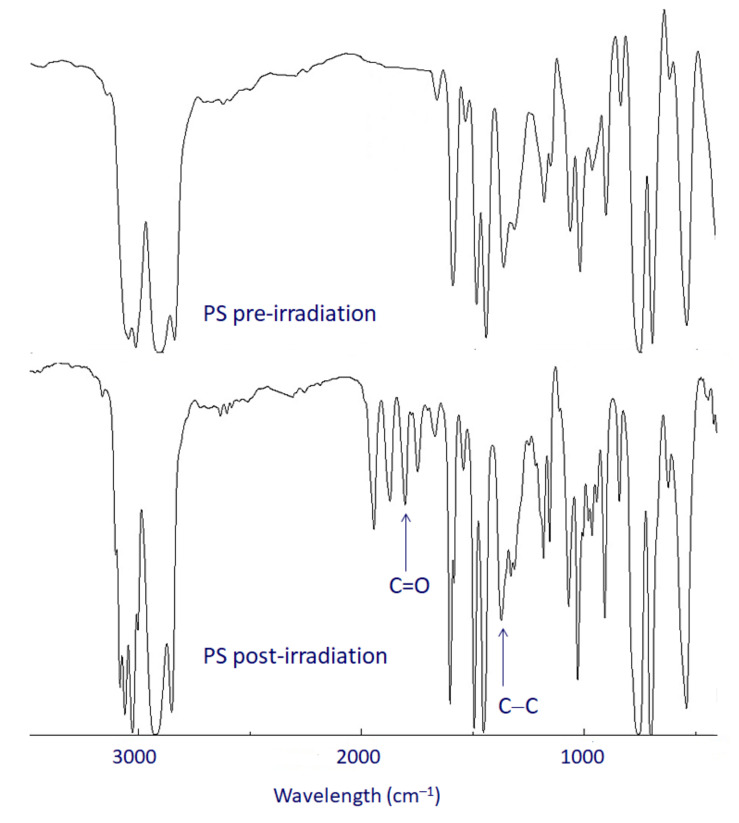
The FTIR spectra of PS film pre- and post-irradiation.

Cephalexin Schiff bases were added at a concentration of 0.5% by weight to the PS, and thin films were prepared (Section 2.3). A UV light was used to irradiate the PS films for a period of time that varied from 50 h to 300 h. The FTIR spectra were recorded pre- and post-irradiation of the film. Figure 3 shows that the intensity of the C=O group absorption band (1730 cm^−1^) has grown significantly after irradiation confirmed photodegradation of the PS film.

**Figure 4:**
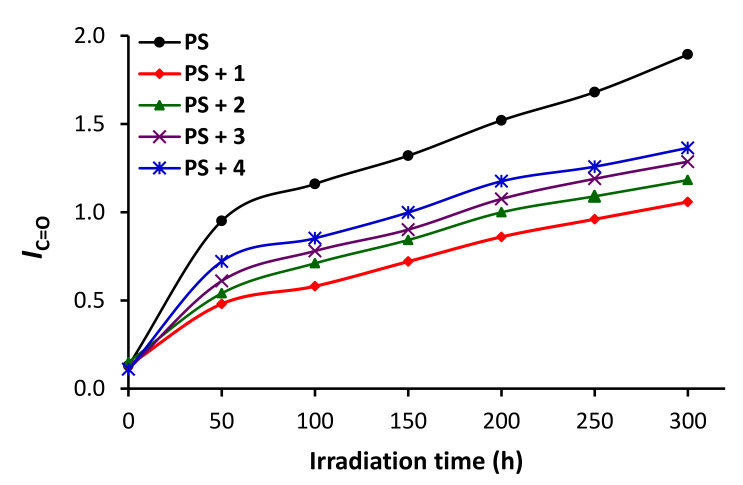
Effect of irradiation on carbonyl index (*I_C=O_*) of PS blends.

The intensity of the C=O group absorbance (*A_C=O_*; 1730 cm^−1^) was monitored during irradiation and compared with that for the C–C bond that appears at 1328 cm^−1^ (*A_C–C_*). The intensity of the C–C bond does not alter during irradiation [40,41]. The carbonyl group index (*I_C=O_*) was calculated using Equation (1) and plotted against time. Figure 4 shows that values of *I_C=O_* rose sharply in the first 50 h and that the *I_C=O_* was significantly higher for the blank PS film compared with the blends containing cephalexin Schiff bases **1**–**4**. For example, the *I_C=O_* was 1.9 for the PS (blank) film after 300 h of irradiation compared with 1.0 for the blend containing Schiff base **1**. Clearly, there was a noticeable decrease in the *I_C=O_* when Schiff bases were used, and it is presumed that this is as a result of direct absorption of UV light through the aromatic rings and heteroatoms of the additives.

*I*_*C*=*O*_ = *A*_*C*=*O*_/*A*_*C*–*C*_ (1)

### 3.4. Effect of UV Irradiation on Weight

**Figure 5:**
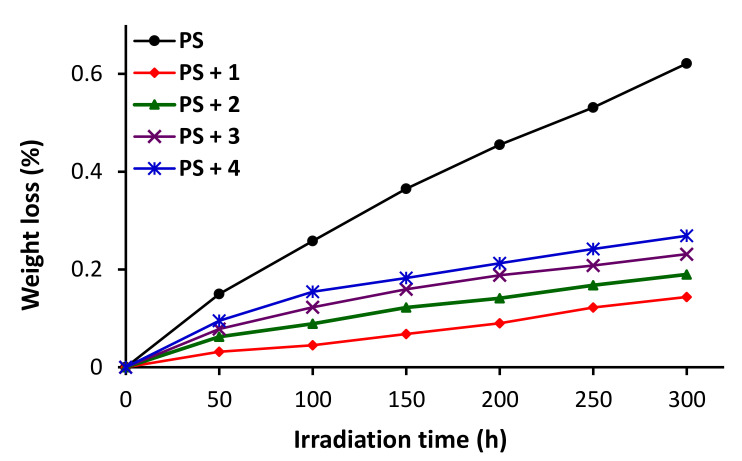
Effect irradiation on the weight loss (%) of PS blends.

Long-term irradiation of PS leads to the formation of free radicals and cross-linking in polymeric chains [42]. As a result, small molecular fragments may be eliminated leading to a decrease in the weight of PS [20]. Therefore, the level of damage taking place can be assessed by observing the weight loss. Equation (2) was used to calculate the percentage weight loss of the sample, from its weight pre (*W_pre_*) and post (*W_post_*) irradiation. The weight loss (%) of PS blends is presented against irradiation time in Figure 5. It is clear that the weight loss is significantly lower in the presence of cephalexin Schiff bases **1**–**4** when compared with the pure film with Schiff base **1** (hydroxyphenyl derivative) providing the most protection to the film. For example, the weight loss percentage at the end of irradiation process was 0.62% for the pure PS film, 0.14% for the PS + **1**, 0.19% for the PS + **2**, 0.23% for the PS + **3**, and 0.27% for the PS + **4**.

*Weight loss (%)* = (*W*_*pre*_ − *W*_*post*_)/*W*_*pre*_ × 100 (2)

### 3.5. Effect of UV Irradiation on Molecular Weight (${\bar{M}}_{V}$)

**Figure 6:**
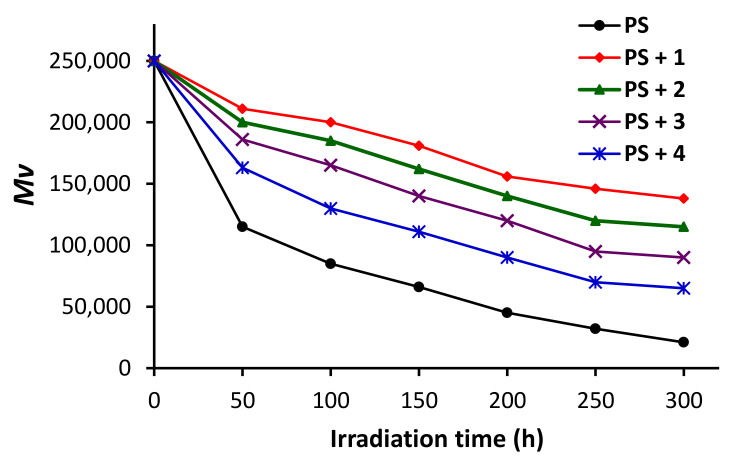
Effect irradiation on the molecular weight of PS blends.

Photodegradation causes a decrease in the average molecular (${\bar{M}}_{V}$) of PS [22]. The degree of the reduction in the ${\bar{M}}_{V}$ is directly proportional to the amount of damage caused by photodegradation. The intrinsic viscosity, [*η*], was measured for the films dissolved in THF after every 50 h of irradiation. The ${\bar{M}}_{V}$ was calculated from [*η*] using Equation (3) [43]. The values of ${\bar{M}}_{V}$ as a function of irradiation time are shown in Figure 6.

(3) $$\left[\eta \right]=1.63\times 10^{-2}\;M_{v}^{0.77}$$

For the pure PS film, the decrease in the ${\bar{M}}_{V}$ was very high in the first 50 h of irradiation having fallen by 54%. After 150 h, the pure PS had lost around 74% of its ${\bar{M}}_{V}$. In contrast, the reduction in the ${\bar{M}}_{V}$ of the PS films containing **1**–**4** was lower. For example, the reduction in the ${\bar{M}}_{V}$. for the PS + **1** blend was approximately 15% after 50 h and 27% after 150 h of irradiation. At the end of irradiation, the ${\bar{M}}_{V}$ was 143,000 for the PS + **1** blend compared with only 21,000 for the blank PS film. The results provide strong evidence that the Schiff bases play an important role in stabilizing the PS film.

### 3.6. Optical Microscopy of PS

**Figure 7:**
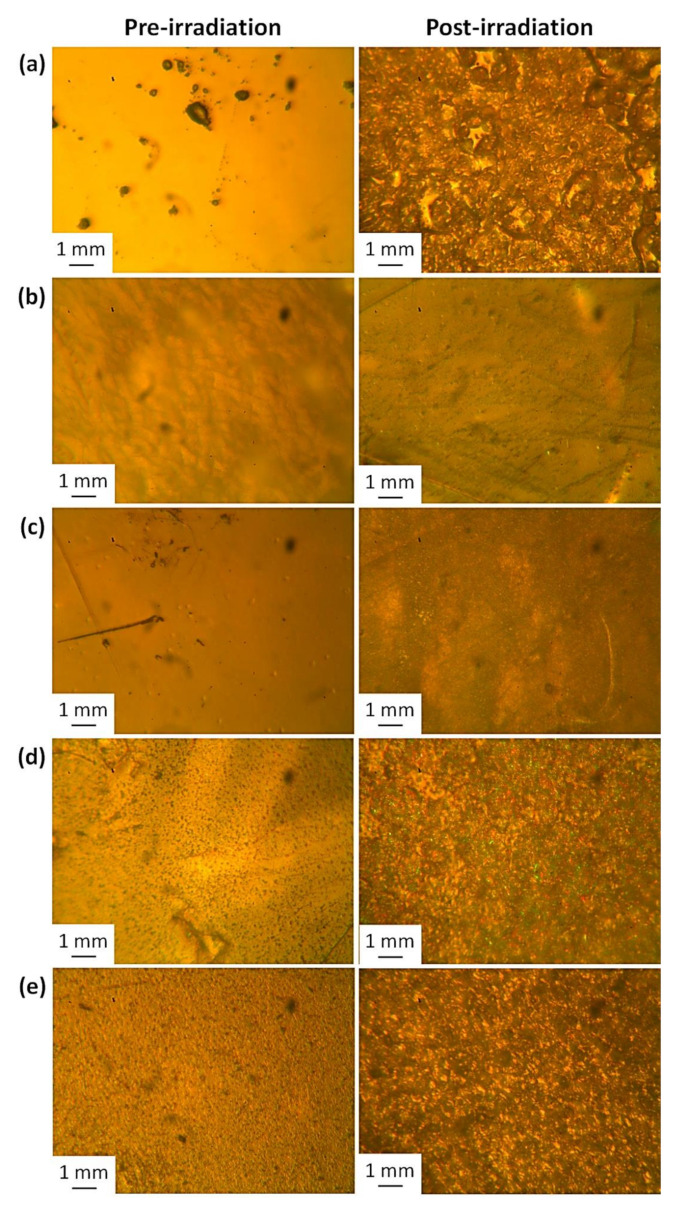
Microscope images of (**a**) PS film, (**b**) PS + **1**, (**c**) PS + **2**, (**d**) PS + **3**, and (**e**) PS + **4** blends, pre- and post-irradiation.

Photodegradation of PS leads to irregularities in the surface and the appearance of defects such as cracks, grooves, and dark spots. These irregularities are mainly due to chain scission and cross-linking and can be assessed using optical microscopy [44,45]. The optical microscopy images (Figure 7) showed that the surfaces of the non-irradiated films were regular with few defects and no cracks or spots. In comparison, the optical image of the irradiated film of pure PS showed the presence of cracks and dark spots. The damage was much less for the PS blends containing cephalexin Schiff bases **1**–**4**. Clearly, cephalexin Schiff bases **1**–**4** improved the photostability PS to a significant degree with **1** and **2** being the most effective as evidenced by their post-irradiation surfaces being regular with few dark spots and cracks. 

### 3.7. FESEM of PS

**Figure 8:**
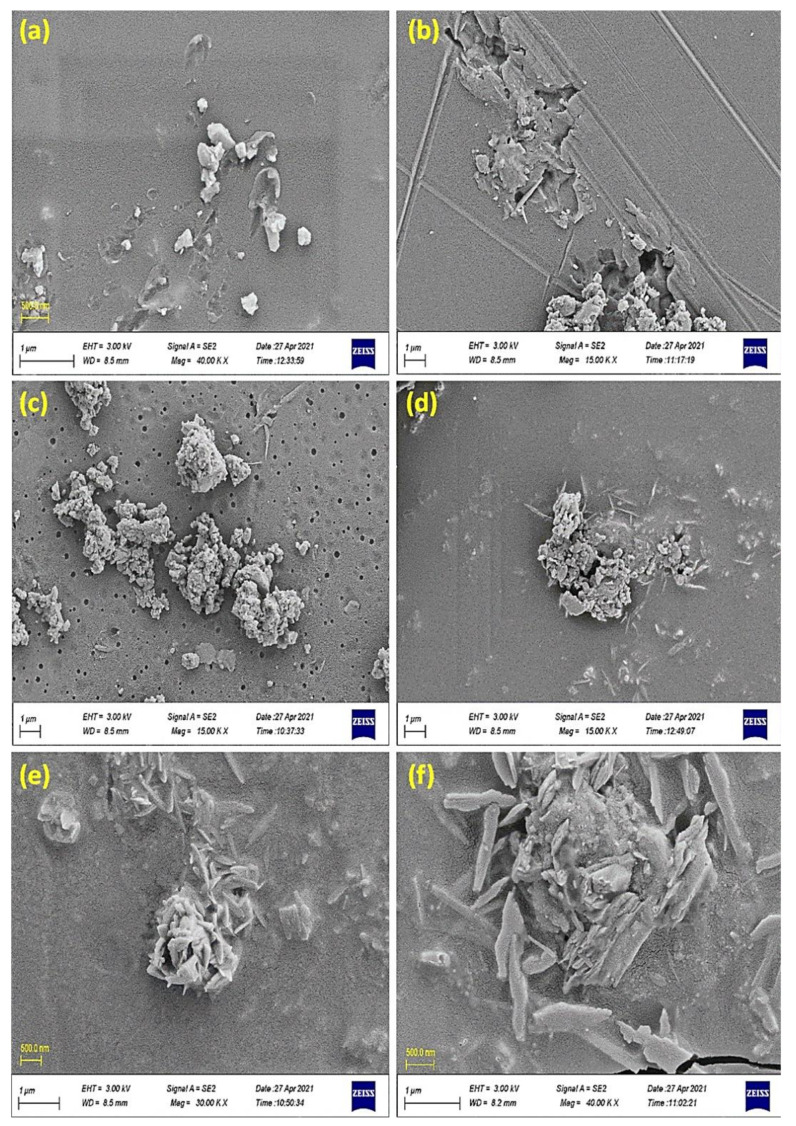
FESEM images of (**a**) PS film pre-irradiation and (**b**) PS, (**c**) PS + **1**, (**d**) PS + **2**, (**e**) PS + **3**, and (**f**) PS + **4** blends post-irradiation.

FESEM is a powerful technique for investigating the damage on the surface of PS as a result of photoirradiation [46,47]. The technique can be used to determine the shape and size of particles, homogeneity, and cross-sections in the samples. The FESEM images for pure PS, recorded pre- and post-irradiation, along with those for the irritated blends containing cephalexin Schiff bases **1**–**4** are shown in Figure 8. The FESEM image for the non-irradiated PS film shows a generally homogeneous, smooth, and regular surface. The surfaces of the irradiated films showed damage post-irradiation, but the defects were more noticeable in the pure film compared with the blends containing cephalexin Schiff bases **1**–**4**. The pore dimensions are 63.6–160.3 nm for the PS film, 34.9–89.1 nm for PS + **1**, 119.3–207.0 nm for PS + **2**, 42.2–267.2 nm for PS + **3**, and 83.0–326.4 nm for PS + **4**.

### 3.8. AFM of PS

**Figure 9:**
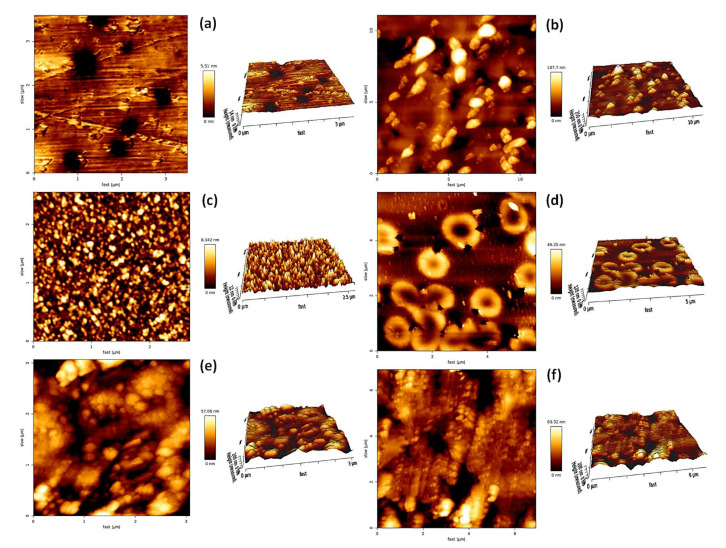
AFM images of (**a**) PS film pre-irradiation and (**b**) PS, (**c**) P S + **1**, (**d**) PS + **2**, (**e**) PS + **3**, and (**f**) PS + **4** blends post-irradiation.

The PS surface was examined further using AFM [48,49]. AFM is used to examine the roughness/smoothness, and homogeneity of a surface. Generally, the non-irradiated materials show smooth surfaces [22]. The AFM images of the PS films are shown in Figure 9. 

The polymeric blends containing Schiff bases have regular and smooth surfaces compared with the blank PS film. However, the AFM images showed black dots in all cases indicating the film’s imperfection. The roughness factor (*Rq*) for the non-irradiated films was very low with very little variation. The *Rq* was 6.2 for the pure PS, 5.4 for PS + **1**, 5.8 for PS + **2**, 6.4 for PS + **3**, and 5.6 for PS + **4**. On the other hand, the *Rq* for the irradiated films was high as 336.2 for the pure PS, 12.4 for PS + **1**, 21.2 for the PS + **2**, 24.4 for the PS + **3**, and 38.3 for the PS + **4**. Clearly, Schiff base **1** has reduced the *Rq* of the PS by 27.1-fold which is very significant (Table 5). Such performance is superior to those obtained with Schiff bases of both 1,2,3,4-triazole-3-thiol [17] and biphenyl-3,3′,4,4′-tetraamine [22]. In addition, cephalexin Schiff bases **1**–**4** provided a reduction in *Rq* much higher than additives used for PVC such as Schiff bases [26,29], tin-complexes [50,51,52,53,54], and polyphosphates [55] (Table 5).

### 3.9. Photostabilization Mechanisms of PS

**Figure 10:**
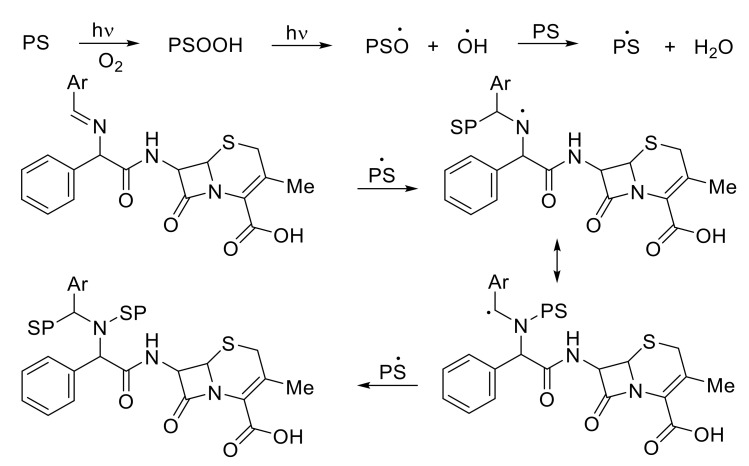
Schiff bases **1**–**4** form stable complexes with PS radicals.

**Figure 11:**
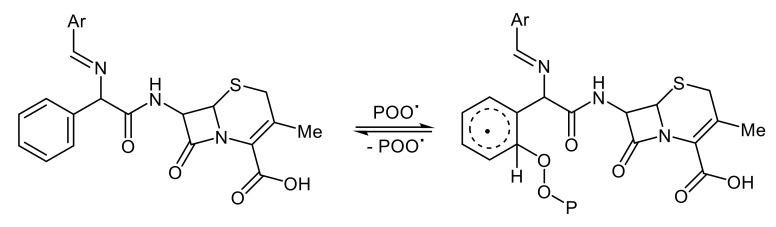
Schiff bases **1**–**4** act as radical scavengers.

**Figure 12:**
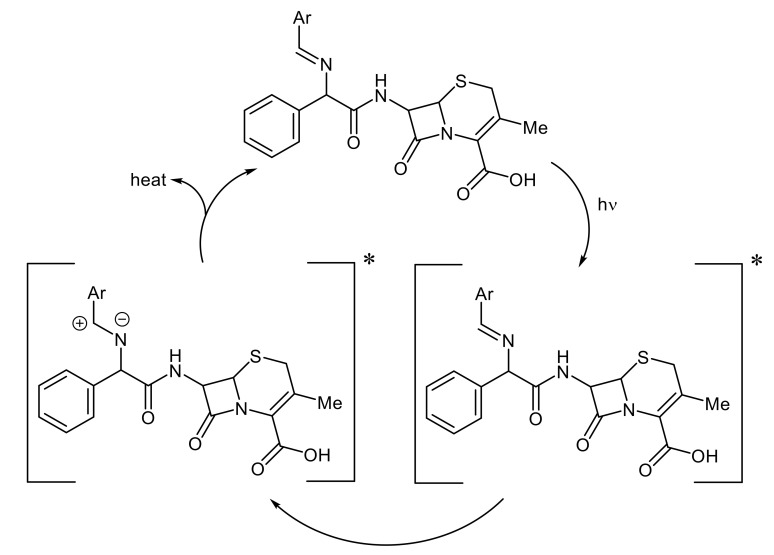
Schiff bases **1**–**4** act as UV absorbers.

Cephalexin Schiff bases **1**–**4** act as good photostabilizers and protected the PS films against photodecomposition when exposed to UV light for a long time. The Schiff bases reduced the level of both photodegradation and chain cross-linking of PS. They are effective because they contain aromatic rings, heteroatoms, and azomethine bonds [56]. Cephalexin Schiff bases can absorb UV light and release the adsorbed energy harmlessly over time (Figure 10, Figure 11 and Figure 12). Schiff base **1** displays the most protection of PS and this is attributed to the extra hydroxyl group that is attached to an aromatic moiety. The hydroxyl group leads to proton transfer followed by an intersystem crossing between the intermediates in the excited state. Such a process leads to the release of the excite-state energy over time at a harmless level for PS [57]. Photooxidation of PS leads to the generation of polymeric chain radicals which can be stabilized through the formation of stable complexes with cephalexin Schiff bases **1**–**4** (Figure 10) [20]. In addition, cephalexin Schiff bases **1**–**4** act as radical scavengers and can destroy high-energy oxygenated species (chromophores) such as peroxide radicals (POO**^.^**). Cephalexin Schiff bases form complexes with peroxide radicals that are highly stable due to resonance within the aromatic rings (Figure 11). The azomethine linkage (CH=N) within the structure of cephalexin Schiff bases plays an important role in PVC photostabilization [31,32]. The CH=N bond enables Schiff bases to act as UV absorbers (Figure 12) [57]. For the potential commercial use of cephalexin Schiff bases as PVC photostabilizers, the ecotoxicological impact of their possible leakage to the surrounding environment should be assessed.

## 4. Conclusions

Several cephalexin Schiff bases were synthesized in high yields and their identities were confirmed by spectroscopy and elemental analysis. In the investigation, the cephalexin Schiff bases acted as efficient polystyrene photostabilizers. Blending them with polystyrene led to a decrease in the formation of small degraded fragments, weight loss, and molecular weight depression upon photoirradiation. In addition, the damage that occurred on the surface of polystyrene was low in the presence of cephalexin Schiff bases when compared with the pure polystyrene film. Moreover, the roughness factor of the polystyrene surface was reduced significantly in the presence of the cephalexin Schiff bases. The cephalexin Schiff bases stabilize polystyrene through aromatic moieties, heteroatoms, and azomethane linkages in the skeleton which act as quenchers for radicals, absorbers for ultraviolet radiation, and form stable complexes with polystyrene high energy species.

## Figures and Tables

**Table 1:** Physical properties and elemental composition of cephalexin Schiff bases **1**–**4**.

Schiff Base	Ar	Color	M.P. (°C)	Yield (%)	Calculated (Found; %)			
					C	H	N	S
**1**	3-HOC_6_H_4_	Yellow	234–235	82	61.19 (61.28)	4.69 (4.72)	9.31 (9.24)	7.10 (7.02)
**2**	4-Me_2_NC_6_H_4_	Brown	201–203	73	62.74 (62.56)	4.48 (4.52)	11.71 (11.62)	6.70 (6.62)
**3**	4-MeOC_6_H_4_	White	222–225	70	61.92 (61.80)	4.98 (5.02)	9.03 (8.96)	6.89 (6.81)
**4**	4-BrC_6_H_4_	Orange	131–132	78	53.70 (53.58)	3.92 (3.95)	8.17 (8.10)	6.23 (6.15)

**Table 2:** Common FTIR absorption bands of cephalexin Schiff bases **1**–**4**.

Schiff Base	OH	CH (Ar)	NH	C=O (Amide)	C=O (Carboxyl)	CH=N	C=C (Aromatic)	C–S
**1**	3489	3041	3207	1759	1693	1568	1546	817
**2**	3464	3059	3271	1768	1683	1620	1564	858
**3**	3477	3039	3272	1758	1693	1587	1569	817
**4**	3320	3041	3288	1768	1728	1643	1541	727

**Table 3:** ^1^HNMR data of cephalexin Schiff bases **1**–**4**.

Schiff Base	^1^H NMR (500 MHz: δ, ppm, *J* in Hz)
**1**	13.13 (s, exch., 1H), 9.63 (s, exch., 1H), 8.64 (s, 1H), 7.66 (s, exch., 1H), 7.52–6.98 (m, 9H), 5.24 (d, *J* = 4.5 Hz, 1H), 5.03 (s, 1H), 4.98 (d, *J* = 4.5 Hz, 1H), 3.41 (d, *J* = 18.0 Hz, 1H), 3.30 (d, *J* = 18.0 Hz, 1H), 2.00 (s, 3H)
**2**	12.60 (s, exch., 1H), 8.89 (s, 1H), 8.60 (s, exch., 1H), 7.75–7.69 (m, 9H), 4.75 (d, *J* = 4.6 Hz, 1H), 4.68 (s, 1H), 4.52 (d, *J* = 4.6 Hz, 1H), 3.45 (d, *J* = 18.2 Hz, 1H), 3.34 (d, *J* = 18.2 Hz, 1H), 3.12 (s, 6H), 1.96 (s, 3H)
**3**	13.19 (s, exch., 1H), 9.31 (s, 1H), 8.61 (s, exch., 1H), 7.46–7.27 (m, 9H), 5.71 (d, *J* = 4.4 Hz, 1H), 5.63 (d, *J* = 4.6 Hz, 1H), 4.97 (s, 1H), 3.49 (d, *J* = 18.4 Hz, 1H), 3.46 (s, 3H), 3.41 (d, *J* = 18.2 Hz, 1H), 2.00 (s, 3H)
**4**	12.82 (s, exch. 1H), 9.00 (s, 1H), 7.92 (s, exch., 1H), 7.75–7.22 (s, 9H), 5.14 (d, *J* = 4.3 Hz, 1H), 4.92 (s, 1H), 4.76 (d, *J* = 4.3 Hz, 1H), 3.27 (d, *J* = 18.3 Hz, 1H), 3.16 (d, *J* = 18.3 Hz, 1H), 1.97 (s, 3H)

**Table 4:** ^13^CNMR spectral data of cephalexin Schiff bases for **1**–**4**.

Schiff Base	^13^C NMR (125 MHz: δ, ppm, *J* in Hz)
**1**	173.7, 171.7, 169.1, 164.2, 143.7, 139.5, 129.0, 128.7, 127.9, 127.4, 126.6, 126.4, 126.2, 118.3, 122.0, 115.6, 79.3, 58.0, 56.9, 30.5, 19.4
**2**	169.7, 164.7, 161.2, 155.0, 151.2, 146.6, 136.2, 129.6, 128.2, 127.5, 126.1, 124.6, 121.3, 105.0, 74.9, 68.3, 56.3, 44.2, 28.0, 20.2
**3**	168.3, 166.7, 163.9, 163.2, 158.0, 138.9, 130.3, 129.1, 128.6, 128.4, 127.8, 126.3, 122.0, 116.6, 73.0, 64.5, 63.5, 55.9, 28.7, 19.3
**4**	173.6, 162.7, 161.1, 158.8, 141.3, 135.9, 132.7, 130.0, 129.2, 128.8, 128.5, 127.6, 126.2, 121.7, 72.4, 62.6, 60.1, 30.5, 20.1

**Table 5:** Fold reduction in the *Rq* for polymeric materials after irradiation.

Polymeric Materials	Additives	Fold-Reduction in *Rq*	Reference
PS	Cephalexin Schiff bases	27.1	Current research
PS	1,2,3,4-Triazole-3-thiol Schiff bases	3.3	[17]
PS	Biphenyl-3,3′,4,4′-tetraamine Schiff bases	8.3	[22]
PVC	Biphenyl-3,3′,4,4′-tetraamine Schiff bases	3.6	[26]
PVC	Melamine Schiff bases	6.0	[29]
PVC	Tin complexes	5.2–16.6	[50,51,52,53,54]
PVC	Polyphosphates	16.7	[55]

## Data Availability

Data are contained within the article.
